# (*E*)-1-[4-(Dimethyl­amino)benzyl­idene]thio­semicarbazide

**DOI:** 10.1107/S1600536808043778

**Published:** 2009-01-08

**Authors:** Yuying Sun, Shizhou Fu, Junhong Zhang, Xiao Wang, Daqi Wang

**Affiliations:** a Analytical and Testing Center of Beihua Univerisity, Jilin 132013, People’s Republic of China; b Department of Chemistry, Liaocheng University, Liaocheng 250059, People’s Republic of China

## Abstract

In the title mol­ecule, C_10_H_14_N_4_S, the thio­rea plane and benzene ring form a dihedral angle of 16.0 (3) Å. In the crystal structure, inter­molecular N—H⋯S hydrogen bonds link the mol­ecules into ribbons extended in the [100] direction; these incorporate inversion dimers.

## Related literature

For the biomedical properties of thio­semicarbazones, see: Beraldo & Gambino (2004 ▶); Bondock *et al.* (2007 ▶). For the crystal structure of the related compound benzyl *N*′-(2-chloro­benzyl­idene)hydrazinecarbodithio­ate, see Shi *et al.* (2008 ▶).
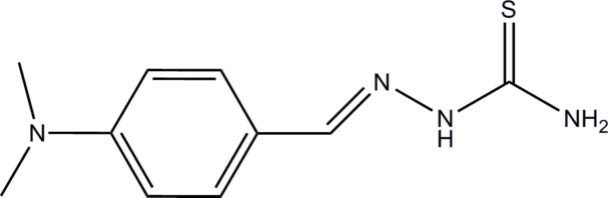


## Experimental

### 

#### Crystal data

C_10_H_14_N_4_S
*M* *_r_* = 222.31
Monoclinic, 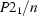

*a* = 5.6984 (13) Å
*b* = 8.9493 (14) Å
*c* = 22.813 (2) Å
β = 93.860 (2)°
*V* = 1160.7 (3) Å^3^
*Z* = 4
Mo *K*α radiation
μ = 0.25 mm^−1^
*T* = 298 (2) K
0.50 × 0.48 × 0.26 mm

#### Data collection

Bruker SMART CCD area-detector diffractometer
Absorption correction: multi-scan (*SADABS*; Sheldrick, 1996 ▶) *T*_min_ = 0.884, *T*_max_ = 0.937
5769 measured reflections
2047 independent reflections
1435 reflections with *I* > 2σ(*I*)
*R*_int_ = 0.037

#### Refinement

*R*[*F* ^2^ > 2σ(*F* ^2^)] = 0.039
*wR*(*F* ^2^) = 0.110
*S* = 1.02
2047 reflections
137 parameters
H-atom parameters constrained
Δρ_max_ = 0.17 e Å^−3^
Δρ_min_ = −0.20 e Å^−3^

### 

Data collection: *SMART* (Siemens, 1996 ▶); cell refinement: *SAINT* (Siemens, 1996 ▶); data reduction: *SAINT*; program(s) used to solve structure: *SHELXS97* (Sheldrick, 2008 ▶); program(s) used to refine structure: *SHELXL97* (Sheldrick, 2008 ▶); molecular graphics: *SHELXTL* (Sheldrick, 2008 ▶); software used to prepare material for publication: *SHELXTL*.

## Supplementary Material

Crystal structure: contains datablocks I, global. DOI: 10.1107/S1600536808043778/cv2492sup1.cif

Structure factors: contains datablocks I. DOI: 10.1107/S1600536808043778/cv2492Isup2.hkl

Additional supplementary materials:  crystallographic information; 3D view; checkCIF report

## Figures and Tables

**Table 1:** Hydrogen-bond geometry (Å, °)

*D*—H⋯*A*	*D*—H	H⋯*A*	*D*⋯*A*	*D*—H⋯*A*
N3—H3*A*⋯S1^i^	0.86	2.84	3.408 (2)	125
N3—H3*B*⋯S1^ii^	0.86	2.57	3.417 (2)	168

Symmetry codes: (i) 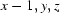; (ii) 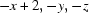.

**Fig. 1.**
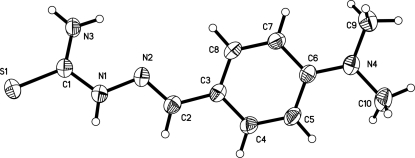
The molecular structure of (I) showing the atomic numbering scheme and 30% probability displacement ellipsoids.

## supplementary crystallographic information

### Comment

The title compound (I) is a derivative of thiosemicarbazones, which are important for drug design (Beraldo & Gambino, 2004) and for synthesis of heterocyclic rings (Bondock *et al.*, 2007).

In (I) (Fig.1), all bond lengths and angles are normal and comparable to those observed in the related compound (Shi *et al.*, 2008). The dihedral angle between the C9/C10/N4 plane and the benzene ring is 10.14 (3) °. The thiorea plane and benzene ring form a dihedral angle of 15.97 (3) °. The C1=S1 bond length of 1.674 (2) Å is intermediate between the values of 1.82 Å for a C—S single bond and 1.56 Å for a C=S double bond. The C=N—N angle in the molecule of 115.69 (19)° is significantly smaller than the ideal value of 120° expected for *sp*^2^ -hybridized N atoms. This is probably a consequence of repulsion between the nitrogen lone pairs and the adjacent N bonds.

In the crystal, the intermolecular N—H···S hydrogen bonds (Table 1) link the molecules into ribbons extended in direction [100].

### Experimental

N,N' –Dimethylaminobenzaldehyde (0.5 mmol), thiosemicarbazide (0.5 mmol) and 10 ml e thanol were mixed in 50 ml flask. After stirring 30 min at 373 K, the resulting mixture was recrystalized from ethanol, affording the title compound as a orange crystalline solid. Elemental analysis: calculated for C_10_H_14_N_4_S: C 54.03, H 6.35, N 25.20%; found: C 54.38, H 6.54, N 25.27%.

### Refinement

All H atoms were placed in geometrically idealized positions (N—H 0.86 and C—H= 0.93–0.96 Å) and treated as riding on their parent atoms, with *U*_iso_(H) = 1.2 *U*_eq_(C,*N*).

### Crystal data

**Table:**

C_10_H_14_N_4_S	*F*(000) = 472
*M**_r_* = 222.31	*D*_x_ = 1.272 Mg m^−^^3^
Monoclinic, *P*2_1_/*n*	Mo *K*α radiation, λ = 0.71073 Å
*a* = 5.6984 (13) Å	Cell parameters from 2093 reflections
*b* = 8.9493 (14) Å	θ = 2.5–25.9°
*c* = 22.813 (2) Å	µ = 0.25 mm^−^^1^
β = 93.860 (2)°	*T* = 298 K
*V* = 1160.7 (3) Å^3^	Block, orange
*Z* = 4	0.50 × 0.48 × 0.26 mm

### Data collection

**Table:**

Bruker SMART CCD area-detector diffractometer	2047 independent reflections
Radiation source: fine-focus sealed tube	1435 reflections with *I* > 2σ(*I*)
graphite	*R*_int_ = 0.037
φ and ω scans	θ_max_ = 25.0°, θ_min_ = 1.8°
Absorption correction: multi-scan (*SADABS*; Sheldrick, 1996)	*h* = −6→6
*T*_min_ = 0.884, *T*_max_ = 0.937	*k* = −10→10
5769 measured reflections	*l* = −27→17

### Refinement

**Table:**

Refinement on *F*^2^	Secondary atom site location: difference Fourier map
Least-squares matrix: full	Hydrogen site location: inferred from neighbouring sites
*R*[*F*^2^ > 2σ(*F*^2^)] = 0.039	H-atom parameters constrained
*wR*(*F*^2^) = 0.110	*w* = 1/[σ^2^(*F*_o_^2^) + (0.0432*P*)^2^ + 0.4004*P*] where *P* = (*F*_o_^2^ + 2*F*_c_^2^)/3
*S* = 1.01	(Δ/σ)_max_ < 0.001
2047 reflections	Δρ_max_ = 0.17 e Å^−^^3^
137 parameters	Δρ_min_ = −0.20 e Å^−^^3^
0 restraints	Extinction correction: *SHELXL97* (Sheldrick, 2008), Fc^*^=kFc[1+0.001xFc^2^λ^3^/sin(2θ)]^-1/4^
Primary atom site location: structure-invariant direct methods	Extinction coefficient: 0.027 (3)

### Special details

**Table:**

Geometry. All e.s.d.'s (except the e.s.d. in the dihedral angle between two l.s. planes) are estimated using the full covariance matrix. The cell e.s.d.'s are taken into account individually in the estimation of e.s.d.'s in distances, angles and torsion angles; correlations between e.s.d.'s in cell parameters are only used when they are defined by crystal symmetry. An approximate (isotropic) treatment of cell e.s.d.'s is used for estimating e.s.d.'s involving l.s. planes.
Refinement. Refinement of *F*^2^ against ALL reflections. The weighted *R*-factor *wR* and goodness of fit *S* are based on *F*^2^, conventional *R*-factors *R* are based on *F*, with *F* set to zero for negative *F*^2^. The threshold expression of *F*^2^ > σ(*F*^2^) is used only for calculating *R*-factors(gt) *etc*. and is not relevant to the choice of reflections for refinement. *R*-factors based on *F*^2^ are statistically about twice as large as those based on *F*, and *R*- factors based on ALL data will be even larger.

### Fractional atomic coordinates and isotropic or equivalent isotropic displacement parameters (Å^2^)

**Table:**

	*x*	*y*	*z*	*U*_iso_*/*U*_eq_	
N1	0.9364 (3)	0.1808 (2)	0.14592 (8)	0.0544 (5)	
H1	1.0442	0.2390	0.1611	0.065*	
N2	0.7454 (3)	0.1467 (2)	0.17756 (8)	0.0497 (5)	
N3	0.7772 (3)	0.0474 (2)	0.06910 (8)	0.0583 (6)	
H3A	0.6548	0.0349	0.0887	0.070*	
H3B	0.7820	0.0093	0.0346	0.070*	
N4	0.0633 (3)	0.1090 (2)	0.39241 (9)	0.0621 (6)	
S1	1.20240 (11)	0.15551 (9)	0.05735 (3)	0.0708 (3)	
C1	0.9570 (4)	0.1249 (2)	0.09195 (9)	0.0473 (5)	
C2	0.7371 (4)	0.2136 (3)	0.22678 (9)	0.0510 (6)	
H2	0.8539	0.2829	0.2374	0.061*	
C3	0.5554 (4)	0.1872 (2)	0.26677 (9)	0.0470 (5)	
C4	0.5549 (4)	0.2699 (3)	0.31838 (10)	0.0560 (6)	
H4	0.6676	0.3441	0.3253	0.067*	
C5	0.3937 (4)	0.2457 (3)	0.35945 (10)	0.0573 (6)	
H5	0.3985	0.3044	0.3932	0.069*	
C6	0.2228 (4)	0.1346 (2)	0.35143 (9)	0.0483 (5)	
C7	0.2210 (4)	0.0514 (3)	0.29886 (9)	0.0528 (6)	
H7	0.1082	−0.0226	0.2916	0.063*	
C8	0.3831 (4)	0.0782 (3)	0.25809 (9)	0.0507 (6)	
H8	0.3771	0.0217	0.2238	0.061*	
C9	−0.1011 (4)	−0.0139 (3)	0.38610 (12)	0.0684 (7)	
H9A	−0.1877	−0.0074	0.3486	0.103*	
H9B	−0.2082	−0.0092	0.4168	0.103*	
H9C	−0.0166	−0.1067	0.3887	0.103*	
C10	0.0832 (6)	0.1853 (3)	0.44832 (12)	0.0900 (10)	
H10A	0.2193	0.1495	0.4711	0.135*	
H10B	−0.0549	0.1664	0.4690	0.135*	
H10C	0.0984	0.2908	0.4419	0.135*	

### Atomic displacement parameters (Å^2^)

**Table:**

	*U*^11^	*U*^22^	*U*^33^	*U*^12^	*U*^13^	*U*^23^
N1	0.0517 (11)	0.0650 (13)	0.0474 (11)	−0.0059 (9)	0.0093 (9)	−0.0067 (10)
N2	0.0506 (10)	0.0566 (12)	0.0425 (10)	0.0063 (9)	0.0089 (8)	0.0035 (9)
N3	0.0458 (10)	0.0862 (15)	0.0438 (11)	−0.0059 (10)	0.0098 (8)	−0.0078 (10)
N4	0.0661 (12)	0.0674 (14)	0.0547 (12)	−0.0014 (10)	0.0186 (10)	−0.0096 (10)
S1	0.0521 (4)	0.0967 (6)	0.0658 (5)	−0.0157 (3)	0.0204 (3)	−0.0145 (4)
C1	0.0442 (12)	0.0545 (14)	0.0433 (12)	0.0065 (10)	0.0034 (9)	0.0047 (10)
C2	0.0608 (13)	0.0469 (13)	0.0454 (13)	0.0028 (11)	0.0048 (11)	0.0003 (10)
C3	0.0582 (13)	0.0433 (13)	0.0396 (12)	0.0080 (10)	0.0041 (10)	0.0000 (10)
C4	0.0701 (15)	0.0447 (13)	0.0541 (14)	−0.0055 (11)	0.0105 (12)	−0.0073 (11)
C5	0.0776 (16)	0.0499 (14)	0.0455 (13)	0.0010 (12)	0.0125 (12)	−0.0134 (11)
C6	0.0521 (12)	0.0480 (13)	0.0450 (13)	0.0087 (10)	0.0042 (10)	−0.0018 (10)
C7	0.0521 (12)	0.0561 (15)	0.0499 (13)	−0.0030 (11)	0.0018 (10)	−0.0084 (11)
C8	0.0583 (13)	0.0545 (14)	0.0389 (12)	0.0055 (11)	−0.0010 (10)	−0.0095 (10)
C9	0.0647 (15)	0.0705 (17)	0.0714 (17)	−0.0004 (13)	0.0154 (13)	0.0057 (14)
C10	0.118 (2)	0.088 (2)	0.0690 (19)	−0.0113 (18)	0.0441 (17)	−0.0195 (16)

### Geometric parameters (Å, °)

**Table:**

N1—C1	1.342 (3)	C4—C5	1.372 (3)
N1—N2	1.380 (2)	C4—H4	0.9300
N1—H1	0.8600	C5—C6	1.396 (3)
N2—C2	1.276 (3)	C5—H5	0.9300
N3—C1	1.315 (3)	C6—C7	1.411 (3)
N3—H3A	0.8600	C7—C8	1.375 (3)
N3—H3B	0.8600	C7—H7	0.9300
N4—C6	1.366 (3)	C8—H8	0.9300
N4—C10	1.445 (3)	C9—H9A	0.9600
N4—C9	1.446 (3)	C9—H9B	0.9600
S1—C1	1.674 (2)	C9—H9C	0.9600
C2—C3	1.445 (3)	C10—H10A	0.9600
C2—H2	0.9300	C10—H10B	0.9600
C3—C8	1.389 (3)	C10—H10C	0.9600
C3—C4	1.391 (3)		
			
C1—N1—N2	121.22 (18)	C4—C5—H5	119.5
C1—N1—H1	119.4	C6—C5—H5	119.5
N2—N1—H1	119.4	N4—C6—C5	121.3 (2)
C2—N2—N1	115.69 (19)	N4—C6—C7	121.8 (2)
C1—N3—H3A	120.0	C5—C6—C7	116.9 (2)
C1—N3—H3B	120.0	C8—C7—C6	121.1 (2)
H3A—N3—H3B	120.0	C8—C7—H7	119.5
C6—N4—C10	120.6 (2)	C6—C7—H7	119.5
C6—N4—C9	121.0 (2)	C7—C8—C3	121.8 (2)
C10—N4—C9	117.4 (2)	C7—C8—H8	119.1
N3—C1—N1	116.51 (19)	C3—C8—H8	119.1
N3—C1—S1	123.55 (17)	N4—C9—H9A	109.5
N1—C1—S1	119.94 (17)	N4—C9—H9B	109.5
N2—C2—C3	123.3 (2)	H9A—C9—H9B	109.5
N2—C2—H2	118.3	N4—C9—H9C	109.5
C3—C2—H2	118.3	H9A—C9—H9C	109.5
C8—C3—C4	116.9 (2)	H9B—C9—H9C	109.5
C8—C3—C2	123.7 (2)	N4—C10—H10A	109.5
C4—C3—C2	119.3 (2)	N4—C10—H10B	109.5
C5—C4—C3	122.3 (2)	H10A—C10—H10B	109.5
C5—C4—H4	118.9	N4—C10—H10C	109.5
C3—C4—H4	118.9	H10A—C10—H10C	109.5
C4—C5—C6	121.1 (2)	H10B—C10—H10C	109.5
			
C1—N1—N2—C2	−175.76 (19)	C9—N4—C6—C5	−175.0 (2)
N2—N1—C1—N3	5.8 (3)	C10—N4—C6—C7	173.9 (2)
N2—N1—C1—S1	−174.31 (15)	C9—N4—C6—C7	5.8 (3)
N1—N2—C2—C3	−177.43 (18)	C4—C5—C6—N4	179.4 (2)
N2—C2—C3—C8	5.5 (3)	C4—C5—C6—C7	−1.4 (3)
N2—C2—C3—C4	−177.3 (2)	N4—C6—C7—C8	−179.8 (2)
C8—C3—C4—C5	0.4 (3)	C5—C6—C7—C8	0.9 (3)
C2—C3—C4—C5	−177.0 (2)	C6—C7—C8—C3	0.2 (3)
C3—C4—C5—C6	0.7 (4)	C4—C3—C8—C7	−0.8 (3)
C10—N4—C6—C5	−6.9 (4)	C2—C3—C8—C7	176.4 (2)

### Hydrogen-bond geometry (Å, °)

**Table:**

*D*—H···*A*	*D*—H	H···*A*	*D*···*A*	*D*—H···*A*
N3—H3A···S1^i^	0.86	2.84	3.408 (2)	125
N3—H3B···S1^ii^	0.86	2.57	3.417 (2)	168

Symmetry codes: (i) *x*−1, *y*, *z*; (ii) −*x*+2, −*y*, −*z*.
